# Guide for Navigating 3D-Printed Cortical Bone Trajectory Screws in Situations with Previously Instrumented Pedicle Screws

**DOI:** 10.1007/s43465-026-01764-8

**Published:** 2026-03-25

**Authors:** Wei-Che Tai, Jen-Chung Liao

**Affiliations:** Department of Orthopedics Surgery, Bone and Joint Research Center, Chang Gung Memorial Hospital, Chang Gung University, No. 5, Fu-Shin Street, Kweishian, Taoyuan, 333 Taiwan

**Keywords:** Adjacent segment disease (ASD), Cortical bone trajectory (CBT), 3D printing, Navigation guide, Pedicle screws

## Abstract

**Purpose:**

Traditional surgical interventions for adjacent segment disease (ASD) repair commonly mandate the exposure of all pre-existing hardware. Cortical bone trajectory (CBT) screws have been innovated to amplify the contact surface between the screw and cortical bone, offering a promising approach to mitigate the challenge sassociated with traditional ASD repair surgeries.

**Methods:**

In this study, a tangible manifestation of the cortical bone trajectory screw navigation guide was produced by utilizing a 3D printer to facilitate the insertion of cortical bone trajectory screws. A comprehensive set of radiographic images and computed tomography scans was gathered to evaluate the accuracy of K-wire through CBT screw guide inside the vertebrae which was previously implanted with a pedicle screw.

**Results:**

The variance in the K-pin circle centre distance before and after surgery ranges from 0.2 mm to 3.54 mm. The K-pin angle difference falls within the range of 0.4° to 5.8° in the sagittal plane, ranges from 1.46° to 1.7° in the transverse plane.

**Conclusion:**

This research process has successfully yielded a navigation guide that holds promise for inserting CBT screw even with previously pedicle screw at the same vertebrae.

## Introduction

Adjacent segment disease (ASD) subsequent to lumbar interbody fusion surgery encompasses pathological alterations occurring in proximity to or at a distance from the mobile segment [1–3]. These pathological changes involve intervertebral disc degeneration, disc herniation, proliferative arthritis of the joints, spinal instability resulting in stenosis, vertebral compression fractures, and loosening of screws [4, 5]. The prevalence of symptomatic ASD varies between 3 and 10 percent, as indicated by different studies [6, 7]. A considerable portion of the individuals affected by ASD symptoms necessitates reoperation. Typically, the surgical intervention for ASD involves decompression and fusion of adjacent spinal segments. Conventional revision procedures often require exposure of pre-existing hardware, which not only induces considerable pain but also elevates the risk of infection and intraoperative blood transfusion [8–10]. Revision surgeries conducted through traditional approaches frequently necessitate the exposure of all pre-existing hardware. This exposure not only induces considerable pain but also elevates the risk of infection and the potential need for blood transfusions during the course of the surgery. Cortical bone trajectory (CBT) screws were devised with the aim of augmenting the contact surface between screws and cortical bone during posterior spinal screw fixation [11, 12]. The unique origin of CBT screws, situated laterally to the transverse process of the articular process, necessitates less muscle dissection [13]. Unlike the lateral-to-medial angle employed in traditional pedicle orbits, CBT screws are inserted at a medial-to-lateral angle. Technical distinctions between CBT and conventional pedicle trajectories enable the utilization of CBT for posterior spinal fixation within a more confined surgical field [14, 15]. This characteristic facilitates the seamless integration of CBT into minimally invasive techniques [16, 17]. Notably, recent advancements in minimally invasive surgery involving CBT screws have been coupled with innovations in patient-specific template guide systems (PST) [18, 19].

Currently, the majority of cases involving lumbar adjacent segment disease (ASD) require re-operation. Typically, this surgical procedure involves exposing pre-existing bone screws to establish connections between adjacent segments, aiming to address the associated symptoms. However, this approach leads to considerable pain and an elevated risk of infection. This study introduces a novel cortical trajectory screw navigation module, designed to enable minimally invasive salvage surgery for lumbar ASD without necessitating the removal of existing pedicle screws. This relatively limited surgical exposure is anticipated to reduce postoperative infection rates, mitigate complications, and shorten recovery. For experimental validation, we employed the SAWBONES lumbar vertebrae simulation model (L1–Sacrum, SKU: 1340–20), which is widely adopted in biomechanical and surgical navigation research for its high anatomical fidelity and standardized material properties, ensuring our results can be reliably compared with prior literature.

## Materials and Methods

### Design of a Navigation Guide for Cortical Bone Trajectory Screws

**Fig. 1:**
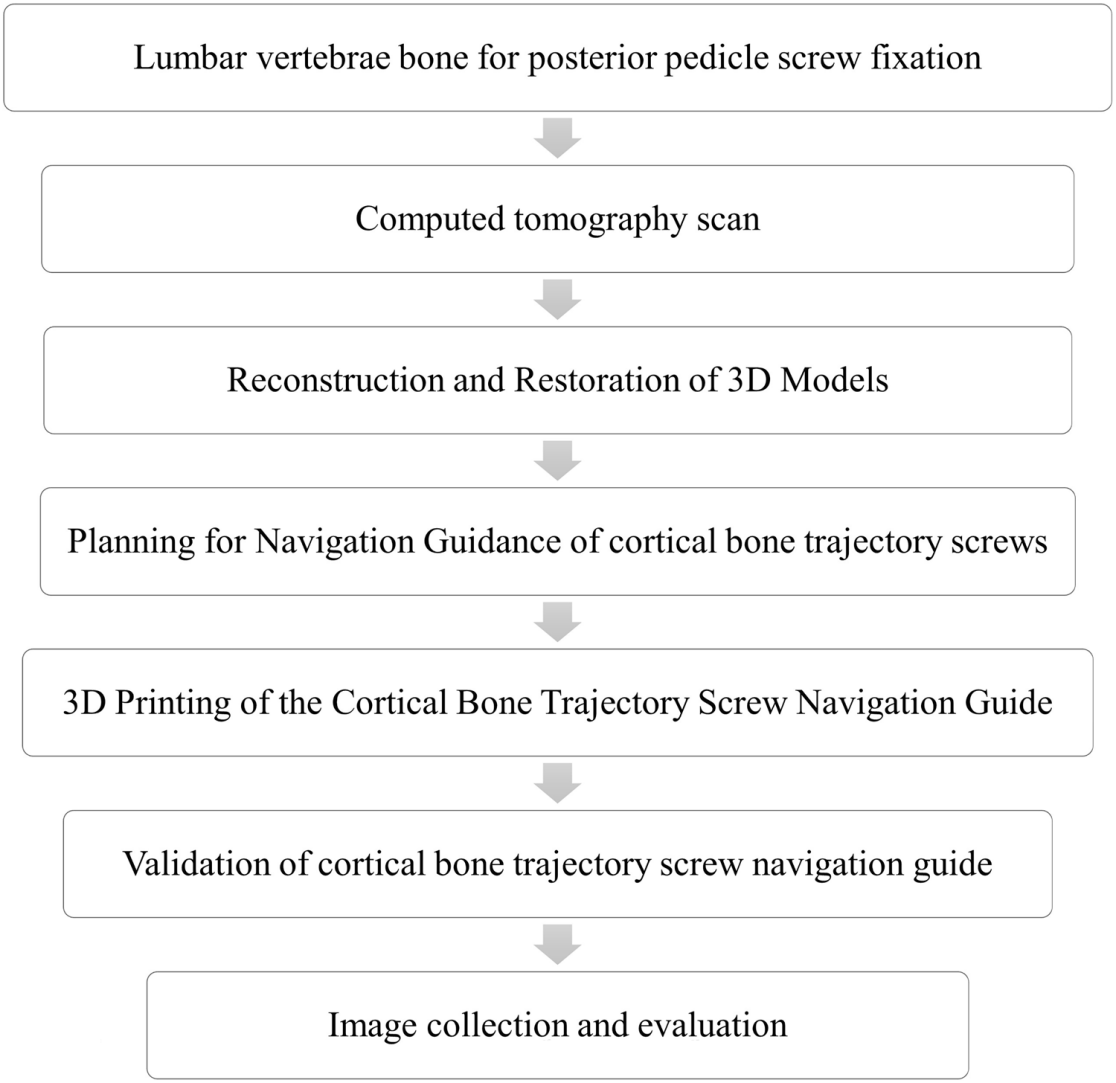
Flowchart for the design of cortical bone trajectory screw navigation guide

The design procedure for the cortical bone trajectory screw navigation guide comprises distinct stages, as illustrated in Fig. 1. Initially, posterior spinal fixation was executed on the simulated lumbar vertebra bone. Subsequently, the open-source software 3D Slicer (The Slicer Community, USA) was employed to reconstruct the three-dimensional model of the simulated lumbar vertebra bone based on the CT scan images. Following this, Shapr3D (Shapr3D Zrt, Hungary) was utilized for the strategic planning of the cortical bone trajectory, determining the entry point and angle for the screw. In the design, considerations were made for the entry point and angle of the screw through the spinous process or connecting rods, ensuring the incorporation of support covers, bridge supports, and screw guide holes to establish a robust and stable structure affixed to the vertebrae. The integration of Boolean operations facilitated a precise fit between the vertebra model and the cortical trajectory screw navigation guide.

### Reconstruction and Restoration of 3D Models for Lumbar Vertebral Specimens

The reconstruction and restoration process of the simulated lumbar vertebrae involved CT scanning, 3D model segmentation, and mesh optimization in preparation for navigation guide design. A lumbar vertebrae simulation bone model (L1–Sacrum, SAWBONES, Spine, Lumbar, L3 to Sacrum, SKU: 1340–20) was used for manual insertion of pedicle screws to simulate posterior fixation. Initial reconstruction was conducted in 3D Slicer to generate detailed anatomical models, followed by post-processing in Meshmixer to ensure surface integrity, correct geometry, and optimize mesh density for subsequent applications.

**Fig. 2:**
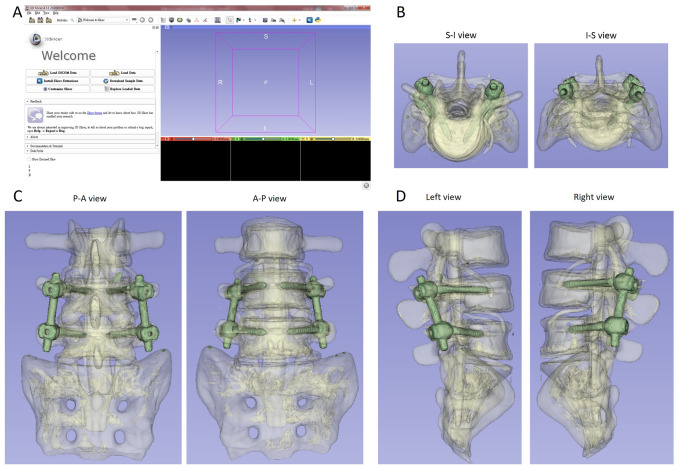
Reconstruction and restoration of 3D models. **A** 3D Slicer reconstruction software was used in this study. **B** 3D models S-I view and I-S view. **C** 3D models P-A view and A-P view. **D** 3D models left view and right view. S-I Superior to Interior, I-S Interior to Superior, P-A posteroanterior, A-P anteroposterior

First, the computed tomography (CT) scan series of the lumbar spine simulated bone in Digital Imaging and Communications in Medicine (DICOM) format for subsequent integration into the 3D Slicer software platform. Utilize the Segment Editor module to initiate the creation of a comprehensive 3D model, encompassing both the pedicle screws and the simulated lumbar spine bone. Set the Threshold range between − 500 and 4000 to accurately delineate the desired structures. Employ the trimming function to eliminate extraneous regions, such as noise surrounding the simulated bones. Initiate the generation of a new segmentation, refining the Threshold range to approximately 1000–4000 to exclusively visualize the pedicle screws within the 3D model window. Employ Logical operators, specifically Boolean operations, on this refined 3D model by combining it with the initially generated 3D model. This process yields a final output devoid of pedicles, presenting a 3D model featuring the lumbar spine simulated bone with integrated screws. Distinguish between the two components by representing them in distinct colors. Upon the completion of the image reconstruction, export the resultant 3D models in STL file format, as illustrated in Fig. 2. This process ensures a comprehensive and detailed representation of the lumbar spine simulated bone and pedicle screws for further analysis or utilization in related applications.

The reconstructed 3D model in STL format was then imported into Meshmixer for further optimization. Employ the "Separate shells" function to delineate non-physically combined objects, resulting in an independent lumbar vertebra simulation bone model. The creation of an autonomous model is essential for ensuring accurate representation and subsequent manipulation. Potential issues may arise from discontinuous broken surfaces within the 3D model, impacting the efficacy of the subsequent navigation module design. To address this concern, employ the “Inspector” tool to rectify any broken surfaces in the 3D model. This step is pivotal in enhancing the overall structural integrity of the model. Subsequently, utilize the “Reduce” function to decrease the number of surfaces in the 3D model. The objective is to achieve a refined model with a reduced mesh surface count, ideally below 200,000 surfaces. This reduction not only streamlines the model but also facilitates smoother processing in subsequent applications. Conclude the process by exporting the refined 3D model to an STL file. This meticulous approach to importing, refining, and exporting ensures the production of a high-quality lumbar vertebra simulation bone model, primed for further analysis or application in various contexts.

### Planning for Navigation Guidance of Cortical Bone Trajectory Screws

**Fig. 3:**
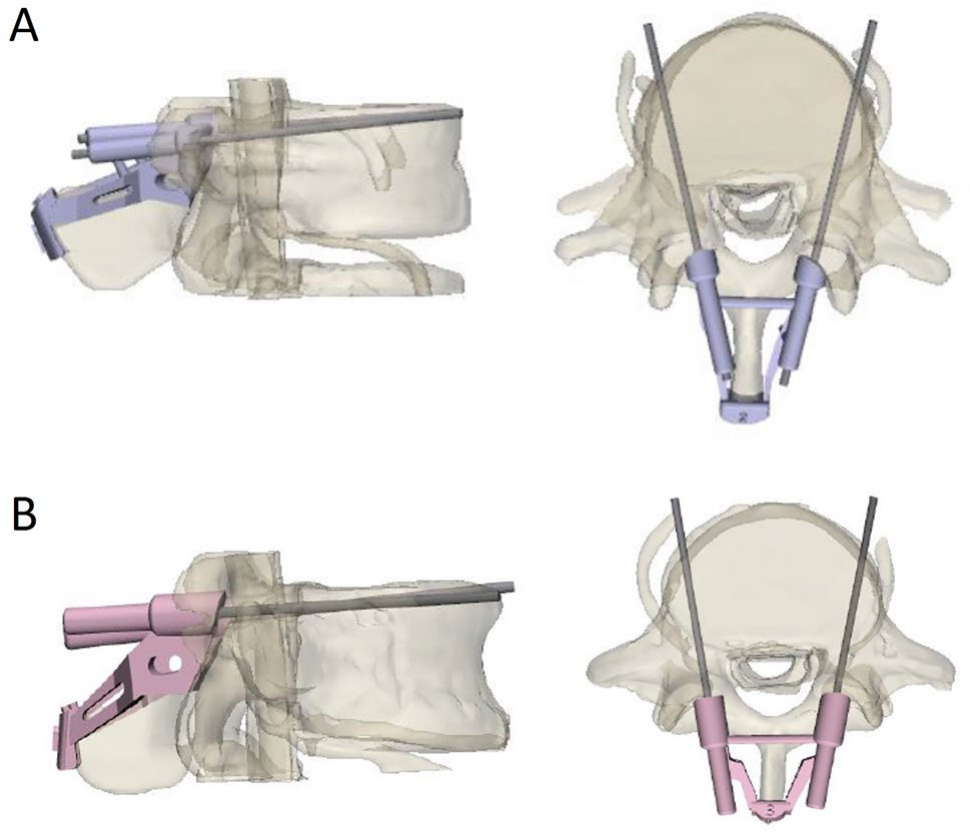
Planning the trajectory for cortical bone trajectory screws. **A** Trajectory of cortical bone trajectory screw: lateral and axial views at lumbar vertebra L4. **B** Trajectory of cortical bone trajectory screw: lateral and axial views at lumbar vertebra L5

**Fig. 4:**
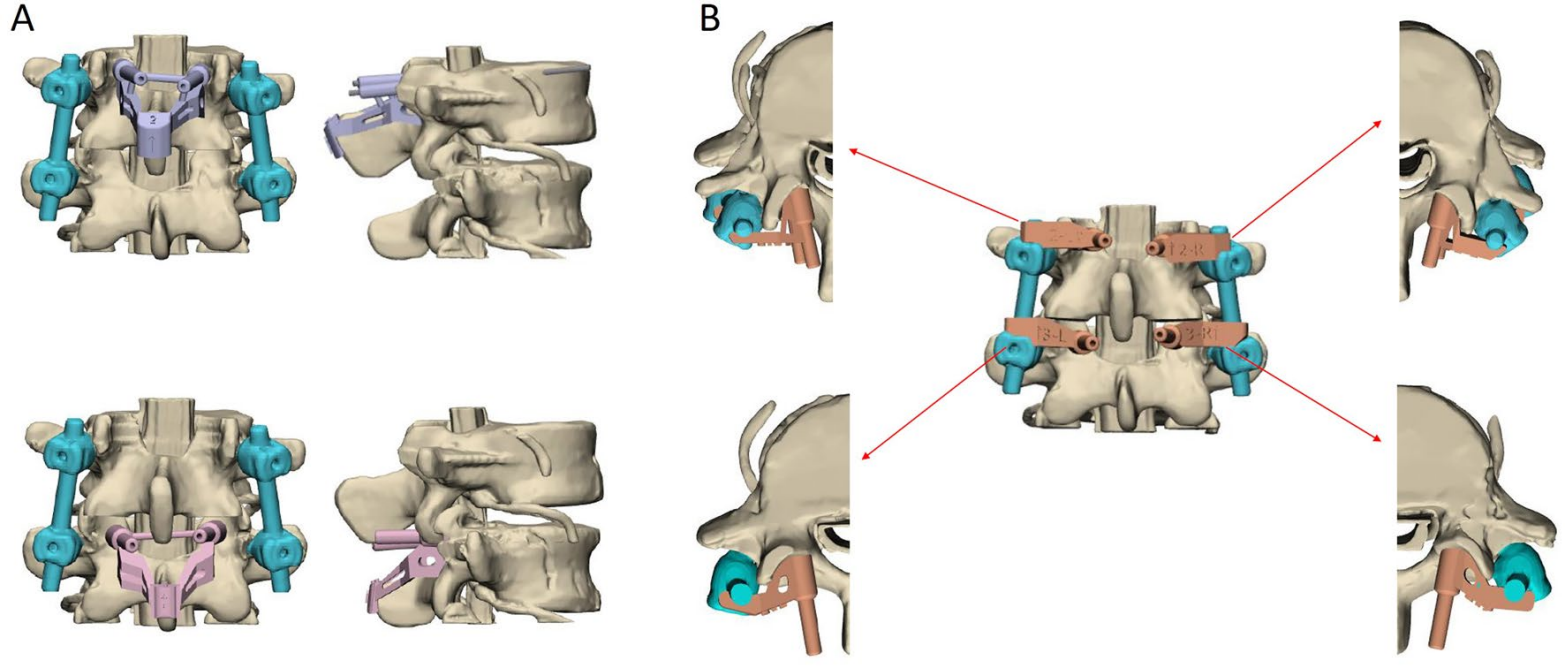
Design of the cortical bone trajectory screw navigation guide. **A** Design for navigation guide with present spinous processes. **B** Design for navigation guide without spinous processes

Initiate the process by importing the rectified STL file into Shapr3D for the purpose of formulating the cortical trajectory screw path. Prescribe specific dimensions for the cortical track screw, specifying a diameter of 5 mm and a length of 40 mm. Execute the trajectory planning for the cortical bone trajectory screws, guiding their passage through the pedicle in the sagittal plane, bottom to top, and in the transverse plane, from medial to lateral. This strategic approach aims to chart a trajectory that avoids interference with the pre-existing pedicle screws, as elucidated in Fig. 3. Evaluate, at this juncture, the spatial adequacy within the vertebral body for the insertion of the cortical trajectory screw (CBT screw). The designed trajectory is pivotal in determining the feasibility of accommodating the CBT screw. Following the trajectory plan, the cortical track screw is coaxially substituted with a K-Pin (3 mm), concurrently generating the guide column, positioning cover, and support system based on the K-Pin's position. These components are amalgamated through a Boolean. Subsequently, apply the Boolean once again to the vertebral body's surface and the pedicle screws, aligning them with the generated cortical trajectory screw navigation guide to ensure a harmonious fit, as depicted in Fig. 4.

### 3D Printing of the Cortical Bone Trajectory Screw Navigation Guide

This study employed an LCD 3D printer (Phrozen Sonic Mini 4 K, TW) for the production of a navigation guide for cortical bone trajectory screws. LCD technology, utilizing liquid crystal display, served as the illuminating mechanism. In this process, the photosensitive resin was exposed to LED light emitted by the LCD display screen, undergoing gradual solidification. Notably, the LCD panel selectively blocked light, allowing illumination only in specific areas. The photosensitive resin underwent layering and successive hardening, resulting in the accumulation of the final product. The STL file of the cortical trajectory screw navigation guide underwent processing through the 3D printing preprocessing software (CHITUBOX). The layer thickness was configured to 0.05 mm, with each layer exposed for 2 to 3 s during the printing phase. The chosen material for printing was a light-curing resin based on Urethane acrylate. Post-printing, a cleansing step with 95% alcohol was implemented to eliminate excess resin from the surface. Subsequently, a secondary curing process ensued, involving exposure to a 405 nm UV light source for a duration of 30 min. This meticulous procedure ensured the fabrication of a precise and functional cortical bone trajectory screw navigation guide.

### Image Collection and Evaluation

The cortical bone trajectory screw navigation guide is employed for the insertion of cortical bone trajectory screws into the simulated lumbar spine following posterior spinal fixation. Subsequent to the insertion, X-ray images are captured, and a computed tomography (CT) scan is conducted. The resulting series of CT scans is then imported into 3D reconstruction imaging software for the purpose of generating a comprehensive 3D image model through reconstruction. Within the 3D model, a simulation of the cortical bone trajectory screw path is executed, and the actual insertion of the cortical bone trajectory screw is facilitated through the guidance of the cortical bone trajectory screw navigation guide. Following the completion of the procedure, the two reconstructed 3D models are superimposed and subjected to comparative analysis. Specifically, the axial deflection angle of the cortical track screw and the deflection angle in the sagittal plane are scrutinized and compared between the two models. This comparative assessment serves as a means to evaluate the precision and effectiveness of the cortical track screw navigation guide in the application of cortical track screws.

## Results

**Fig. 5:**
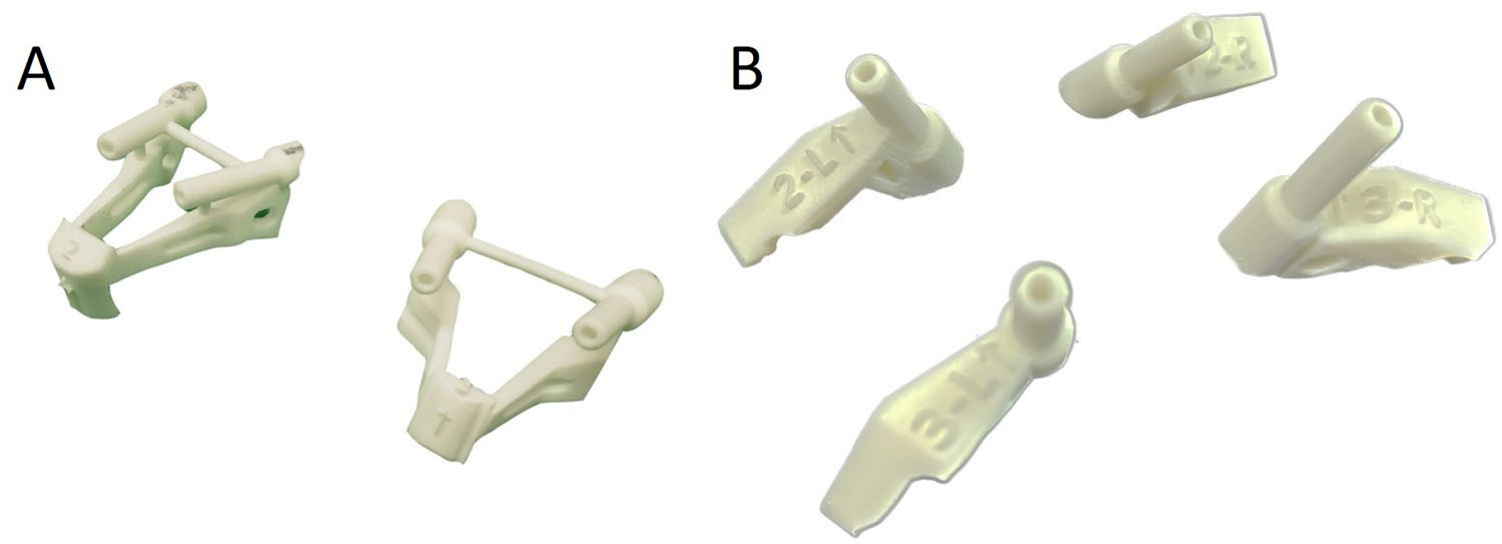
Three-dimensional Printing of the navigation guide for cortical bone trajectory screws. **A** Navigation guide with present spinous processes. **B** Navigation guide without present spinous processes

**Fig. 6:**
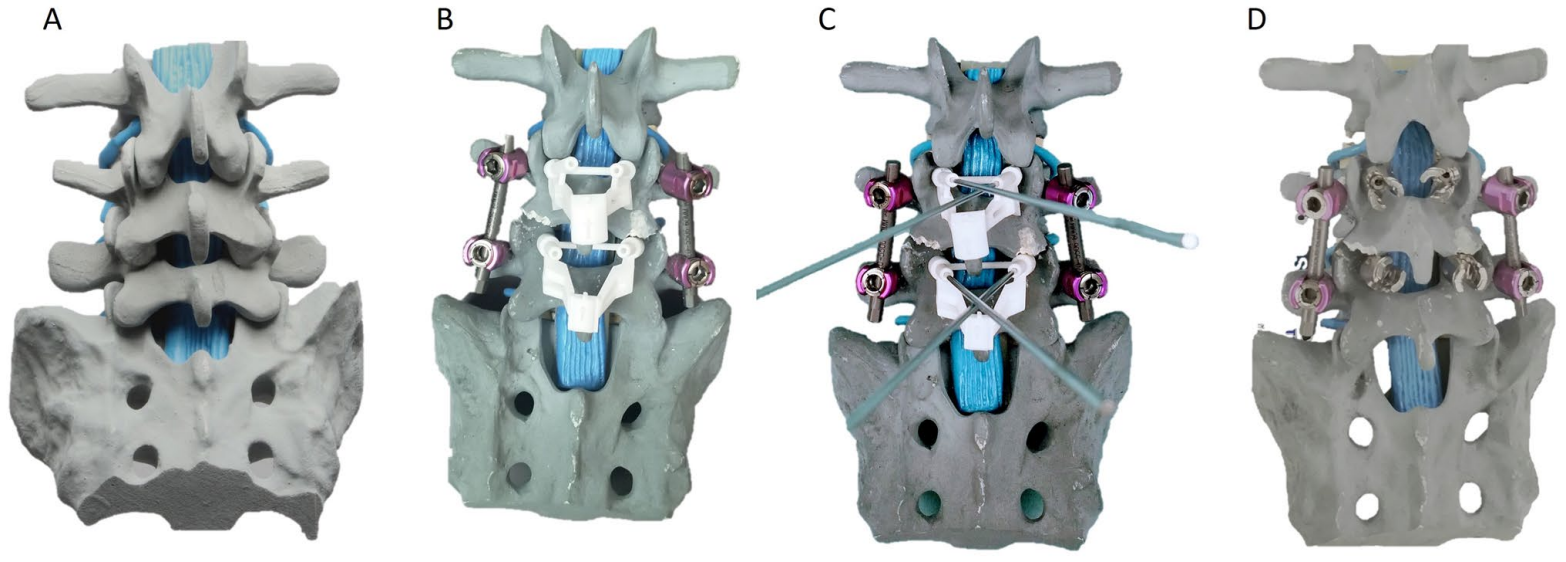
Utilizing three-dimensional printing technology, a navigation guide for cortical bone trajectory screws was produced alongside a simulated lumbar spine model. This ensemble serves to illustrate the sequential process of implanting cortical bone trajectory screws. **A** Lumbar spine simulated bone model. **B** Placement of Cortical Bone Trajectory Screw Navigation Guide onto lumbar spine. **C** Insertion of Kirschner Wire. **D** Extraction of the Cortical Bone Trajectory Screw Navigation Guide with the Kirschner wire in place for tapping and subsequent screw placement

**Fig. 7:**
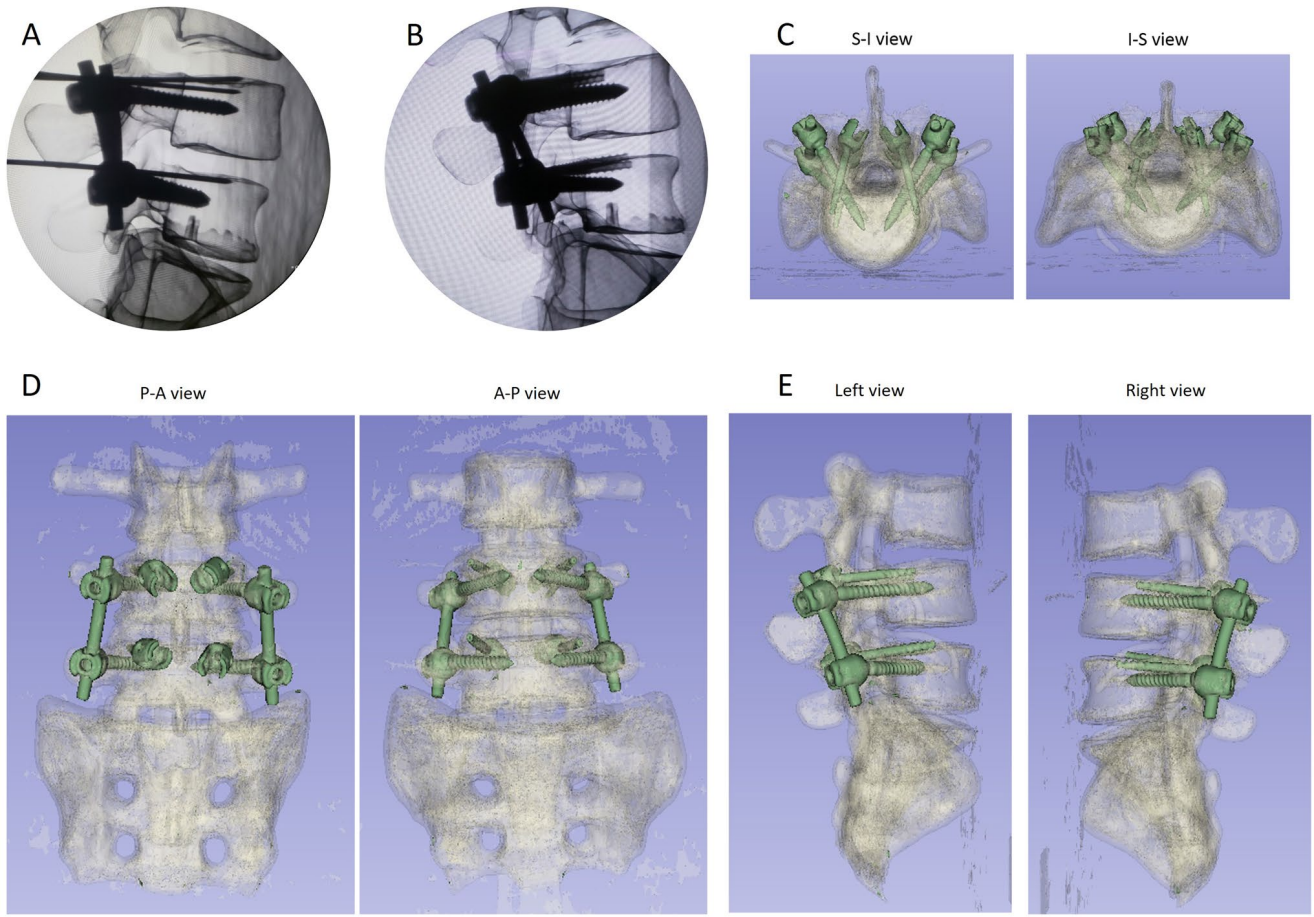
Reconstruction and restoration of 3D models post-implantation of cortical bone trajectory screws. **A** Lateral view following Kirschner wire insertion. **B** Lateral view post-implantation of cortical bone trajectory screws. **C** 3D models S-I view and I-S view. **D** 3D models P-A view and A-P view. **E** 3D models Left view and right view. S-I Superior to Interior, I-S Interior to Superior, P-A posteroanterior, A-P anteroposterior

**Fig. 8:**
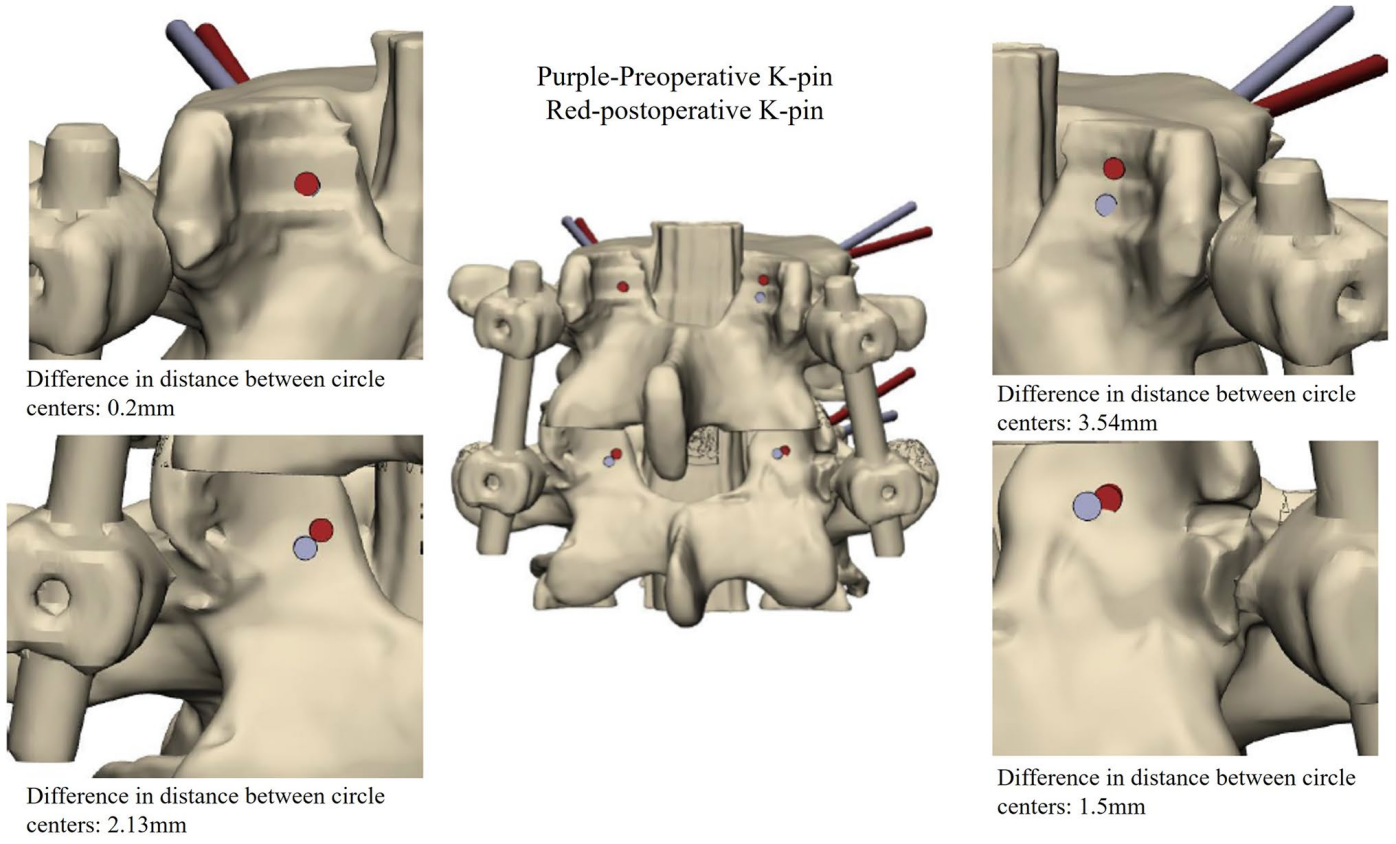
Comparison chart of circle center distances: the discrepancy in the upper left quadrant is 0.2 mm, while the lower left quadrant exhibits a difference of 2.13 mm. Moving to the upper right quadrant, there is a 3.54 mm difference, and in the lower right quadrant, the discrepancy is measured at 1.5 mm

**Fig. 9:**
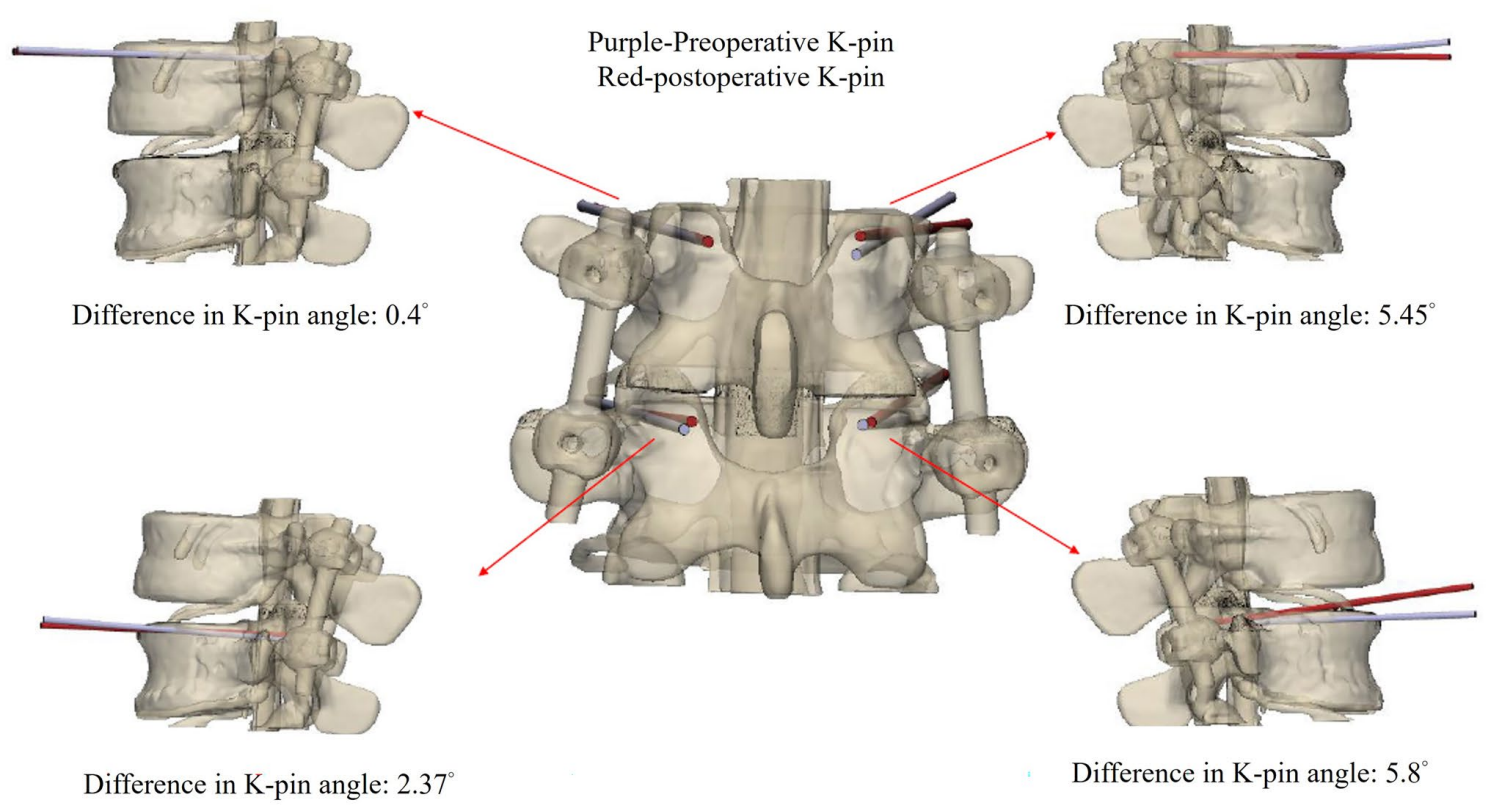
Comparison of angles in the sagittal plane: the disparity in the upper-left quadrant is 0.4 degrees, in the lower-left quadrant is 2.37 degrees, in the upper-right quadrant is 5.45 degrees, and in the lower-right quadrant is 5.8 degrees

**Fig. 10:**
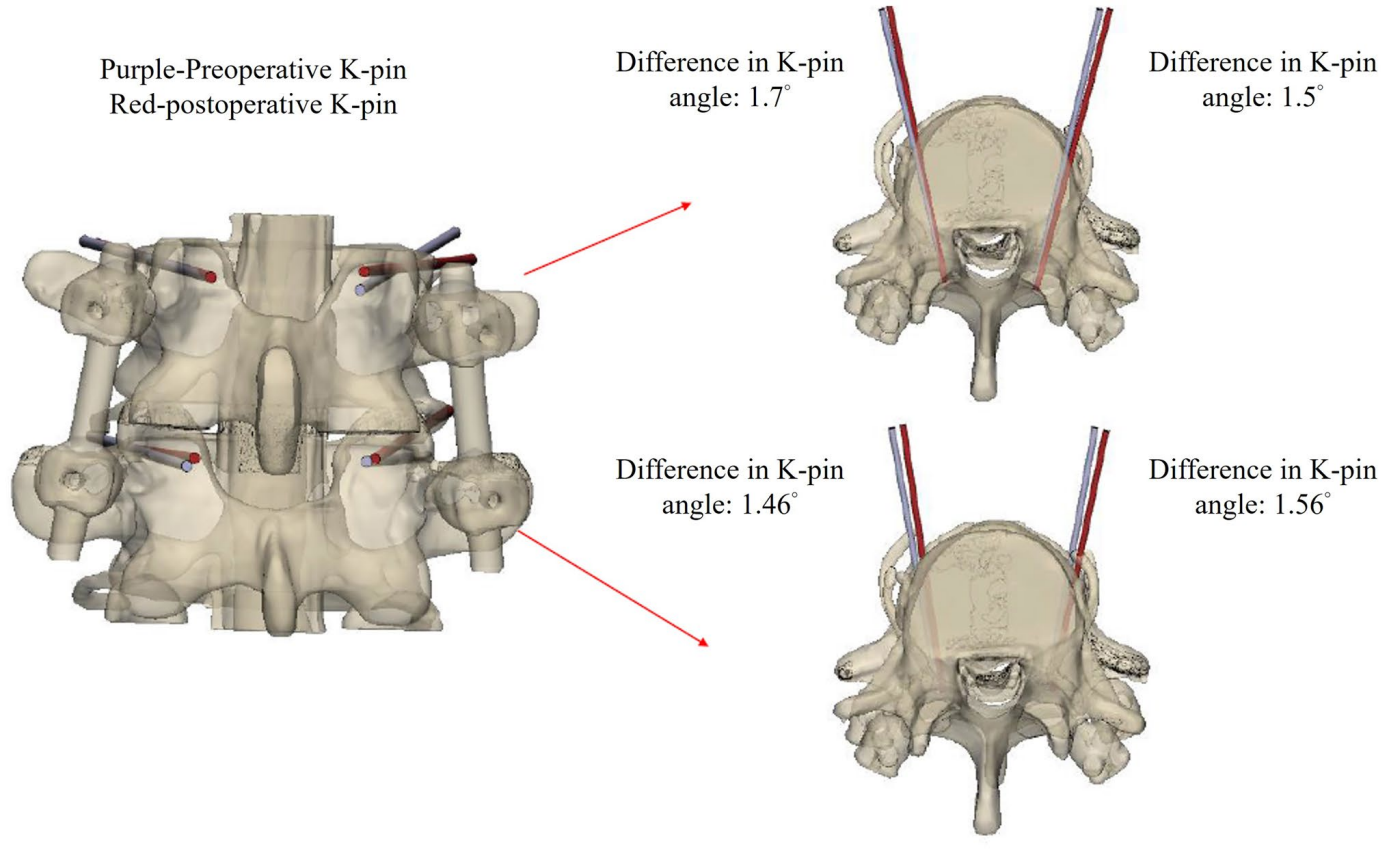
Comparative analysis of cross-sectional view angles: the discrepancy in the upper-left quadrant measures 1.7°, in the lower-left quadrant is 1.46°, in the upper-right quadrant is 1.5°, and in the lower-right quadrant is 1.56°

In this study, we developed two separate navigation guides utilizing 3D printing technology. One guide was specifically designed for situations where the spinous process is present, while the other was tailored for scenarios where the spinous process is absent, as illustrated in Fig. 5. The guides were affixed to the lumbar simulated bone, followed by K-pin insertion and confirmation using C-arm imaging. After the acquisition of imaging data, cortical bone trajectory screws were meticulously inserted, as illustrated in Fig. 6. The ensuing CT scan series was imported into 3D reconstruction imaging software to facilitate the creation of a detailed 3D image reconstruction, as illustrated in Fig. 7. This reconstructed 3D model was then manipulated to replicate the actual cortical bone trajectory and the path of screw insertion, thereby generating a postoperative K-pin model. The preoperative and postoperative K-pin models were superimposed, allowing for the measurement of the center distance of the screw entry point and angles in each direction to ascertain accuracy. The disparity in the center distance of the nail entry point before and after surgery ranged between 0.2 and 3.54 mm, as elucidated in Fig. 8. When considering the sagittal plane, the angle difference of the pin was observed to be within the range of 0.4° to 5.8°, as depicted in Fig. 9. Additionally, the pin angle difference from the cross-sectional view was determined to be between 1.46° and 1.7°, as illustrated in Fig. 10. This comprehensive evaluation provides insights into the precision and reliability of the designed navigation guides and the associated surgical procedures.

## Discussion

In previous studies, the predominant discourse on screw navigation guides primarily centered around the initial surgical fixation involving a single set of screw navigation guides. This emphasis is evident in works such as that of Fei Guo et al. in 2017 [20], Jiwon Kim et al. in 2019 [21], and Bhavuk Garg in 2019 [22]. While scholars like Keitaro Matsukawa and others in 2020 [18] and Andrew Kanawati and colleagues in 2021 [23] did, on occasion, explore the development of navigation guides for the simultaneous drilling of two sets of screws, as exemplified by the work of Yu-Wen Tseng et al. in 2018 [24], explored dual-screw guide systems, the present study uniquely addresses postoperative salvage surgery, filling a gap in the literature. The precision evaluation outcomes reveal variations in the K-pin circle center distance ranging from 0.2 to 3.54 mm before and after surgery. Analyzing from the sagittal plane, the observed K-pin angle disparity falls within the range of 0.4° to 5.8°. When considering the cross-sectional view, the K-pin angle discrepancy ranges between 1.46° and 1.7°. These findings highlight the most substantial deviation occurring in the sagittal plane. This discrepancy is attributed to the inherent slight elasticity of the K-pin. Particularly when drilling into a spine with elevated hardness, deformation transpires due to thrust, leading to a displacement of the drilling point. To mitigate such deviations in needle entry points, it is recommended that future designs of screw navigation guides incorporate adjustments in the length and thickness of the guide string. Increasing these parameters will serve to diminish K-pin deformation during drilling, consequently averting angle deviations at the needle entry point. This insight informs potential refinements in the design of navigation tools for improved accuracy in spinal procedures.

Importantly, the cortical bone trajectory screw navigation guide developed in this study offers significant advantages in reducing infection risk during revision surgery. By enabling precise screw placement without the need to remove pre-existing pedicle screws, the approach minimizes surgical exposure and tissue dissection. This reduced invasiveness decreases the risk of soft tissue infection and preserves local immune and vascular function. Additionally, avoiding hardware removal limits intraoperative contamination and mechanical disturbance of existing implants, which are potential sources of postoperative infection. Collectively, these factors contribute to enhancing surgical safety and improving postoperative recovery outcomes.

Additionally, while this study demonstrated the feasibility and accuracy of the cortical bone trajectory screw navigation guide in simulated bone models, future work will focus on clinical validation to confirm its applicability, safety, and efficacy in real-world surgical scenarios. Such clinical trials are essential to establish the navigation guide’s performance under complex anatomical variations and operative conditions, ultimately supporting its translation into routine spinal surgery practice.

Furthermore, this study is limited by its reliance on simulated bone models, which cannot fully reproduce the complexity of intraoperative anatomy, soft-tissue constraints, and real-time surgical variability. While the findings demonstrate the feasibility and accuracy of the cortical bone trajectory screw navigation guide under controlled conditions, its applicability, safety, and efficacy in real-world surgical scenarios remain unverified. Future work will therefore require cadaveric validation and prospective clinical trials to evaluate the guide’s performance under complex anatomical variations and operative conditions, ultimately determining whether it can be reliably translated into routine spinal surgery practice.

## Conclusion

This article elucidates the design methodology of the navigation guide, employing three-dimensional model image reconstruction and computer-aided design techniques. The utilization of simulated bone and cortical bone trajectory screw navigation guides facilitates the emulation of surgical procedures aimed at addressing lumbar adjacent segment lesions. Through the research process detailed in this study, the CBT navigation guide developed in this study represents an innovative approach for lumbar ASD salvage procedures, demonstrating accurate screw implantation in simulated vertebrae.
